# Supplementary material for: Grb2 phosphorylation antagonizes EGFR-driven ras activation by interfering with condensate assembly
Source: PLoS One. 2026 Oct 26;21(10):e0361303. doi: 10.1371/journal.pone.0361303 (PMC13704879; doi:10.1371/journal.pone.0361303)
Supplement: Supplementary file 1 [file pone.0361303.s013.docx]

**Supplemental Tables**

| Sequence | Modifications | Charge | MH + [Da] | m/z [Da] | Note |
| --- | --- | --- | --- | --- | --- |
| VLNEEcDQNWYK | C6(Carbamidomethyl) | 2 | 1597.69658 | 799.35193 | Tyr 37 |
| VLNEEcDQNWyKAELNGK | C6(Carbamidomethyl);  Y11(Phospho) | 2 | 2289.98516 | 1145.49622 | Tyr 37 |
| VLNEEcDQNWYKAELNGK | C6(Carbamidomethyl) | 3 | 2210.01670 | 737.34375 | Tyr 37 |
| NyIEMKPHPWFFGK | Y2(Phospho) | 3 | 1873.84989 | 625.28815 | Tyr 52 |
| NYIEMKPHPWFFGK |  | 2 | 1793.88445 | 897.44586 | Tyr 52 |
| DIEQVPQQPTyVQALFDFDPQEDGELGFR | Y11(Phospho) | 3 | 3461.55979 | 1154.52478 | Tyr 160 |
| DIEQVPQQPTYVQALFDFDPQEDGELGFRR |  | 3 | 3537.69577 | 1179.90344 | Tyr 160 |
| DIEQVPQQPTYVQALFDFDPQEDGELGFR |  | 3 | 3381.59543 | 1127.87000 | Tyr 160 |
